# The Evolutionary Misfit: Evolution, Epigenetics, and the Rise of Non-Communicable Diseases

**DOI:** 10.3390/epigenomes9040051

**Published:** 2025-12-13

**Authors:** Stefano Amatori

**Affiliations:** Department of Biomolecular Sciences, University of Urbino Carlo Bo, 61032 Fano, PU, Italy; stefano.amatori@uniurb.it

**Keywords:** evolutionary medicine, evolutionary mismatch, non-communicable diseases, epigenetic plasticity, epigenetic inheritance, aging, urban health

## Abstract

Human life expectancy has risen dramatically in the last century, but this demographic triumph has come at the cost of an explosion of non-communicable diseases (NCDs), threatening the sustainability of healthcare systems in aging, low-fertility societies. Evolutionary medicine provides a framework to understand, at least in part, this paradox. Many vulnerabilities to disease are not failures of design but the predictable outcomes of evolutionary trade-offs, constraints, and mismatches. Evolutionary mismatch theory explains how traits once advantageous in ancestral environments become maladaptive in modern contexts of abundance, sedentarism, and urbanization. The developmental origins of health and disease (DOHaD) concept describes how epigenetic plasticity in early life can buffer or amplify these mismatches, depending on whether adult environments align with developmental forecasts. Transgenerational epigenetic inheritance, even if still debated in humans, may further influence phenotypic plasticity, increasing or mitigating the mismatch. In evolutionary terms, the theories of mutation accumulation, antagonistic pleiotropy, and the disposable soma explain why longer lifespans, and ecological and social conditions profoundly different from those in which we developed, increase the likelihood that these costs are expressed clinically. Because most NCDs can be prevented and effectively controlled but not cured, efforts should prioritize quality of life for people, families, and communities. At the individual level, aligning lifestyles with evolved biology can mitigate risk, but the greatest leverage lies in population-level interventions. Urban health strategies represent a forward-looking attempt to realign modern environments with human biology. In this way, the concept of the evolutionary misfit becomes not just a diagnosis of maladaptation, but a guide for building healthier, more sustainable societies.

## 1. Introduction: Evolutionary Medicine and the “Misfit” Concept

Evolutionary medicine emerged in the early 1990s with the seminal article ‘The Dawn of Darwinian Medicine’ by Randolph Nesse and George Williams [1,2]. The central claim is that many human vulnerabilities to disease are not design flaws, but the predictable consequences of evolutionary compromises, trade-offs, and historical constraints. Proximate explanations (the physiological and pathological mechanisms) are complemented by ultimate explanations that ask why selection has produced bodies sufficient for reproduction yet suboptimal for long-term health [3].

Our species evolved under ecological and social conditions profoundly different from those we now inhabit. Metabolic, immune, and neurobehavioral systems were shaped in contexts of energetic variability and high habitual physical activity [4,5,6], dense microbial exposure and environmental biodiversity [7,8,9,10], and robust day–night cycles that tightly entrained circadian physiology [11,12]. Today these systems confront nutritional abundance and ultra-processed food environments [13], sedentarism [4], sanitized and low-diversity ecologies [7,8,9], and pervasive artificial light at night [11,12]. In addition, the nutritional, epidemiological, and demographic transitions triggered by the Industrial Revolution dramatically reshaped human ecology and biology, producing substantial changes in life history traits such as age and size at maturity, patterns of fertility, and lifespan [14]. In this view, Homo sapiens appears “misfit” not because of intrinsic fragility, but because cultural and technological change has far outpaced genetic evolution, creating an adaptive lag between biological design and modern environments [15,16]. A classic example is the evolution of lactase persistence, which arose in pastoralist populations only after the adoption of dairying practices. Even in this relatively simple adaptive context, genetic evolution has been incomplete and geographically uneven, illustrating how cultural innovations can create novel selective environments faster than biology can respond [3,15]. The framework of niche construction describes how humans continuously modify their environments in ways that reshape selection pressures. Through agriculture, urbanization, technological innovation, and social organization, humans have become not only products but also architects of their selective landscape. In this sense, the diseases of civilization are the biological costs of our own ecological success, manifestations of bodies optimized for Pleistocene ecologies but now inhabiting anthropogenic environments of unprecedented novelty and abundance [14,15].

The historical roots of this idea are older than the field itself. In the 1960s, Neel’s “thrifty genotype” hypothesis proposed that alleles favoring efficient energy storage, adaptive under famine, became liabilities in modern abundance [17]. While influential, this hypothesis has been criticized for oversimplifying human evolutionary history, overestimating the frequency and selective impact of famines, and neglecting the polygenic architecture of metabolic traits. In parallel, the notion of an “environment of evolutionary adaptedness” in psychology framed behavior as calibrated to ancestral ecologies [18]. Despite limitations of the original thrifty genotype model, these lines of reasoning converged into what is now termed evolutionary mismatch theory, a cornerstone for evolutionary medicine [19,20].

**Figure 1:**
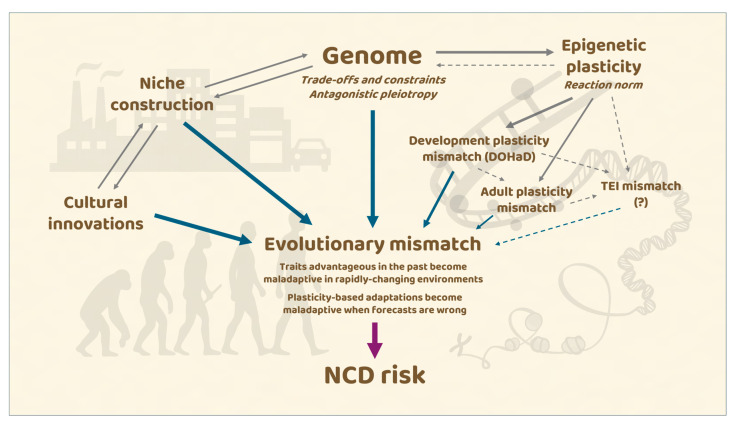
Conceptual framework illustrating how evolutionary, cultural, and epigenetic processes jointly contribute to non-communicable disease (NCD) risk. The model integrates multiple levels of causation: genetic constraints, trade-offs, and antagonistic pleiotropy; cultural innovations and human-driven niche construction; developmental plasticity, adult plasticity and the still debated transgenerational epigenetic inheritance (TEI), including the limits imposed by reaction norms. These pathways converge to generate evolutionary mismatch when ancestral adaptations become maladaptive in rapidly changing environments or when developmental forecasts fail to match adult conditions. Arrow thickness reflects the relative importance of each process. Dashed arrows indicate connections that are not yet fully established in the literature.

Mismatch, however, is plural. Authors commonly distinguish evolutionary mismatch, where traits once advantageous become maladaptive under novel environmental conditions; and developmental mismatch, as framed by the developmental origins of health and disease (DOHaD) paradigm, in which early-life cues set physiological trajectories that prove costly when adult conditions diverge [21,22,23]. A third emerging dimension is the possibility of transgenerational mismatch, whereby environmentally acquired epigenetic states in parents could become maladaptive when transmitted to offspring developing in markedly different ecologies [24,25]. These layers collectively manifest as physiological and behavioral dysregulation, such as chronic stress activation, circadian disruption, or maladaptive lifestyle preferences. Figure 1 offers an integrated conceptual framework illustrating how niche construction, cultural innovation, evolutionary trade-offs, and epigenetic plasticity interact across the life course to shape susceptibility to non-communicable diseases. The model synthesizes the main themes discussed in the review and outlines the multilevel pathways through which environmental change can translate into biological vulnerability.

It should be noted that this framework is applied well beyond aging and non-communicable diseases. Host–pathogen interactions are inherently coevolutionary, and immune systems tuned for high pathogen loads can become dysregulated when sanitation, antibiotics, and vaccines transform exposure landscapes; under these conditions, immune calibration may overshoot into allergy and autoimmunity [26,27,28,29]. Closely related is the human microbiota, historically shaped by diverse diets, environmental microbes, and intergenerational transmission. Rapid shifts due to cesarean delivery, formula feeding, antibiotics, urban living, and ultra-processed foods impoverish microbial diversity and function, with downstream effects on immune education and metabolism that can be interpreted through a mismatch lens [30,31,32,33,34,35]. Cancer, too, fits this logic: tissue architectures and proliferative programs that promoted growth, repair, and reproduction entail “trade-offs,” especially in long-lived organisms, so that longer lifespans, endocrine milieus, and novel carcinogenic exposures unmask oncogenic risk rooted in earlier adaptations [36,37,38,39,40]. Finally, mental disorders have been a particularly active arena for mismatch hypotheses. Systems governing stress reactivity, sociality, and reward—calibrated for small, cooperative groups and punctuated threats—can misfire amid chronic psychosocial novelty, status dynamics, and digital hyper-stimulation [2,41,42,43,44,45,46]. Although many of these topics lie outside the main scope of the present review, they underscore the breadth of the mismatch framework across evolutionary medicine.

At the same time, important caveats remain within the framework of evolutionary mismatch. For example, there was no single ancestral environment to which humans were perfectly adapted: our past is a mosaic of habitats and lifeways [20]. There is also the risk of “paleofantasy,” the romanticized assumption that ancestral life was uniformly healthier [47,48]. Finally, cultural evolution (medicine, agriculture, urbanization) can both mitigate and exacerbate mismatches, often faster than genetic evolution can respond [3].

Against this backdrop, the review proceeds to examine how evolutionary and developmental mismatches converge to shape today’s burden of NCDs, how trade-offs and aging theories rationalize vulnerability across the life course, and how individual strategies and, especially, urban policies might help realign modern life with human biology.

## 2. Trade-Offs and Constraints: Why Bodies Are Not Optimal, but Good Enough

A fundamental premise of evolutionary medicine is that natural selection does not design perfect bodies but rather favors phenotypes that are “good enough” to secure reproductive success in a given environment [3]. The apparent imperfections of the human body, often revealed by its susceptibility to disease, can be explained through the concept of evolutionary trade-offs. In simple terms, when selection enhances one trait, it often does so at the expense of another, because organisms operate under constraints of energy, physiology, and ecology [49,50,51].

The principle of antagonistic pleiotropy, first articulated by Williams in 1957, is a clear illustration. A genetic variant may confer a benefit early in life—such as increased fertility or stronger immune responses—yet prove detrimental later, contributing to senescence, autoimmunity, or cancer [19,52]. From this perspective, vulnerabilities like osteoporosis, Alzheimer’s disease, or certain cancers are not evolutionary mistakes, but rather the late-life costs of traits that enhanced reproductive fitness earlier in life.

Constraints are not only genetic but also developmental and historical. Human bipedalism, for example, freed the hands for tool use and favored endurance running, symbolizing a key adaptation, but it also produced a narrow birth canal that increases obstetric risks [53,54,55]. Similarly, the design of the retina, with photoreceptors placed behind layers of neurons, is not optimal for vision but is the product of evolutionary history and developmental pathways that precluded alternative arrangements [56,57,58]. Such imperfections reflect what Stephen Jay Gould and Richard Lewontin famously called the “spandrels” of evolution, by-products of adaptation and constraint rather than direct targets of selection [59].

Many trade-offs relevant to modern health involve the immune system and metabolism. Strong inflammatory responses have been crucial for pathogen defense, but in contemporary low-pathogen environments they predispose to chronic inflammatory and autoimmune diseases [26,27,28,29,60]. Similarly, energy allocation that favored fat storage in unpredictable environments of scarcity now drives the epidemics of obesity and type 2 diabetes in societies of abundance [17,61,62,63]. Even psychological traits reveal trade-offs: anxiety and heightened vigilance may have improved survival in hostile ancestral settings but today manifest as generalized anxiety disorders when hyper-activated in safe, resource-rich contexts [64].

The broader lesson is that evolution works through local optima, not universal perfection. Selection maximizes reproductive fitness in specific contexts, balancing benefits and costs across life stages and environments. As highlighted by Stearns and Medzhitov, organisms (including humans) are “jury-rigged” solutions to ecological challenges, and their traits must always be understood in light of trade-offs and constraints [3]. Disease thus becomes more intelligible not as a sign of failure, but as a predictable by-product of evolutionary logic.

## 3. Aging, Evolutionary Theories and the Rise of Non-Communicable Diseases

### 3.1. Evolutionary Theories of Aging

One of the central insights of evolutionary biology is that aging, or senescence, is not a programmed process designed to benefit the species, but rather a by-product of how natural selection operates. Indeed, it is widely recognized that selection acts most strongly on traits that influence reproductive success. Once reproduction has occurred, the force of selection diminishes, allowing deleterious mutations or physiological decline to accumulate [3,65].

Several evolutionary theories have been proposed to explain this process. The mutation accumulation hypothesis, first formulated by Medawar, posits that harmful mutations expressed late in life are weakly selected against, simply because most individuals in ancestral environments did not live long enough for those mutations to matter [66]. Williams expanded this reasoning with the theory of antagonistic pleiotropy, according to which the same genes may have beneficial effects early in life but deleterious effects later, contributing to degenerative diseases [52]. Finally, the disposable soma theory of Kirkwood highlights the fundamental trade-off between reproduction and somatic maintenance: resources invested in producing and raising offspring inevitably come at the cost of long-term repair and survival [67].

Recent work unifies these perspectives in a hierarchical framework that maps genes, molecular pathways, organismal traits, vital rates, and fitness, clarifying how different mechanisms generate senescence [68]. In this view, the disposable soma theory is a physiologically explicit case of antagonistic pleiotropy (allocation to reproduction vs. maintenance), while the developmental theory of aging (DTA) explains senescence as age-specific mis-optimization of gene expression (late-life hyperfunction or hypofunction of pathways tuned for early development) arising via antagonistic pleiotropy or via mutation accumulation under weak late-age selection [68]. This synthesis preserves the classical logic yet provides testable predictions about which pathways should show allocation costs versus regulatory mistuning.

### 3.2. Aging Before and After the Transition to Modernity

Contrary to a widespread belief, aging was already present in Homo sapiens long before the demographic transition associated with agriculture, sanitation, and medicine. Paleodemographic evidence indicates that even in small-scale hunter-gatherer populations, a fraction of individuals survived into their 60s or 70s, experiencing the physiological declines characteristic of senescence [3]. What modernity altered was not the existence of aging but its prevalence. Sharp reductions in infant and maternal mortality, together with hygiene, antibiotics, and vaccination, extended average lifespan and reshaped population age structures. As argued by Corbett and colleagues, this shift allowed many more individuals to reach ages at which chronic, late-onset conditions become common [14].

### 3.3. The Evolutionary Origins of Age-Related Disease: Mechanisms and Frameworks

The evolutionary theories of aging provide a powerful scaffold for interpreting the contemporary surge of age-related pathology. Rather than mere “wear and tear,” chronic diseases often reflect delayed costs of traits that enhanced earlier fitness. Robust early-life inflammatory responses, advantageous against infection, predispose later to atherosclerosis and neurodegeneration—a paradigmatic case of antagonistic pleiotropy underlying “inflammaging” [69,70]. Likewise, pathways that promote growth and reproduction can increase oncogenic risk in extended lifespans.

Mechanistic biogerontology has converged on shared hallmarks of aging—including genomic instability, telomere attrition, epigenetic alterations, loss of proteostasis, mitochondrial dysfunction, and deregulated nutrient sensing—that vary across tissues, individuals, and species [71,72,73]. The hierarchical framework proposed by Lemaître et al. [68] clarifies that aging arises from two broad classes of evolutionary mechanisms: (i) resource allocation trade-offs between reproduction and somatic maintenance, and (ii) age-specific mis-regulation of pathways originally optimized for early development. The key implication is that age-related diseases emerge either because maintenance is under-prioritized early in life, or because developmental programs continue to operate when they should be downregulated, processes whose consequences become amplified in modern environments and longer lifespans [68,74,75].

### 3.4. Longevity, Mismatch, and the Modern Burden of NCDs

As life expectancy increased dramatically in the twentieth and twenty-first centuries, the burden of non-communicable diseases rose in parallel [76]. From an evolutionary perspective, modern longevity widens the temporal window over which late-acting liabilities—whether from mutation accumulation, antagonistic pleiotropy, disposable-soma allocation, or DTA mis-regulation—can express clinically [72,73]. Superimposed on this is evolutionary mismatch: pathways tuned for early growth, reproduction, and acute defense now operate for decades in obesogenic, circadian-disruptive, and low-pathogen environments, exposing costs that selection could not efficiently remove [3,20]. In this light, cancer, cardiometabolic disease, and neurodegeneration are not random failures but expected late-life consequences of evolutionary design under novel ecological conditions [68,71].

## 4. Developmental Plasticity, Epigenetic Inheritance, and Mismatch

**Figure 2:**
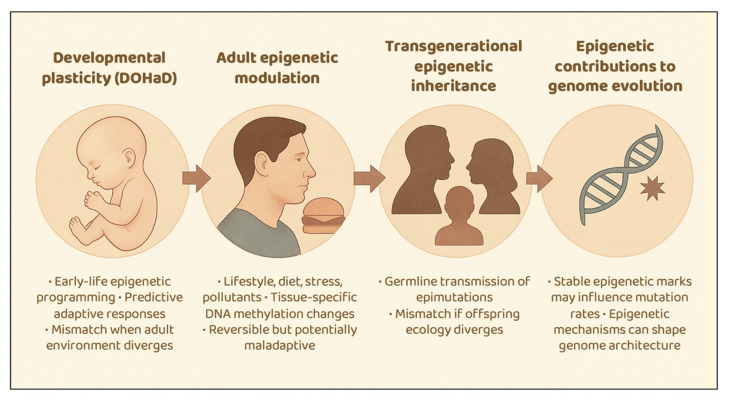
Epigenetic plasticity and mismatch. Environmental exposures can shape epigenetic marks at multiple life stages, with implications for health and evolutionary trajectories. Developmental plasticity (DOHaD) involves early-life epigenetic programming that may misalign with adult environments. Adult exposures—such as diet, stress, and pollutants—can induce reversible epigenetic changes that may become maladaptive. Transgenerational epigenetic inheritance (TEI), though still debated in humans, reflects the transmission of parental epigenetic marks that may mismatch offspring ecologies. Beyond plasticity, stable epigenetic modifications may contribute to genome evolution by influencing mutation rates, transposon activity, and selection, adding a long-term dimension to mismatch dynamics.

Epigenetic mechanisms provide multiple layers of responsiveness to environmental variability across the life course. Early-life developmental plasticity calibrates physiology to predicted conditions, while adult epigenetic modulation allows more reversible responses to lifestyle and environmental exposures. Transgenerational epigenetic inheritance (TEI) may transmit some effects across generations, and epigenetic influences on genome evolution operate over longer timescales. Together, these processes intersect with evolutionary mismatch theory, highlighting both the adaptive potential and the limits of plasticity in shaping health and disease (Figure 2).

### 4.1. Developmental Plasticity and the DOHaD Paradigm

Developmental plasticity is one of the most fundamental strategies through which organisms, including humans, adapt to their environment. During fetal and early postnatal life, signals such as maternal nutrition, endocrine status, or psychosocial stress act as predictive cues for the ecology into which the individual will be born. This flexibility is mediated by epigenetic mechanisms, including DNA methylation, histone modifications, and non-coding RNAs, that regulate gene expression without altering nucleotide sequences [77,78,79,80,81,82].

The developmental origins of health and disease (DOHaD) framework formalizes these insights, describing how early-life exposures calibrate growth, metabolism, and physiology. When adult environments match developmental forecasts, the outcome may be adaptive. However, when forecasts are violated, the result is developmental mismatch and increased vulnerability to disease. Classic epidemiological evidence comes from the Dutch Hunger Winter, where individuals exposed to famine in utero developed persistent metabolic alterations and, when later living in abundance, showed increased risk of obesity, diabetes, and cardiovascular disease [79,83,84].

Similar dynamics apply to maternal stress, which can program hypothalamic–pituitary–adrenal (HPA) axis hyperactivity, advantageous in harsh ecologies but a liability in modern contexts of psychosocial stability [85,86,87]. Thus, developmental plasticity provides essential flexibility, but it also carries the risk of maladaptation when environments change too abruptly or fall outside historical ranges.

One final question naturally arises: why is developmental plasticity unable to compensate for evolutionary mismatch, even when the environment of birth does not differ from that of adulthood? The crux lies in the inherent limits of plasticity, encapsulated in the concept of the “reaction norm” [88,89,90]. A reaction norm describes the range of phenotypes that a given genotype can produce across different environments, defining the pattern and limits of its environmentally induced plastic responses. When the environment diverges beyond the historic conditions that shaped these reaction norms, plastic adjustments fall short and mismatch ensues.

Differently from early-life exposures, epigenetic responses to environmental exposures in adulthood are generally considered less pervasive and more reversible. Nevertheless, accumulating evidence shows that adult lifestyle factors, including diet, physical activity, psychosocial stress, and exposure to pollutants, can still reshape the epigenome in tissue-specific ways [91,92,93]. Epigenetic clocks are DNA methylation–based estimators of biological age that integrate age-sensitive CpG sites whose methylation patterns reflect cumulative molecular changes driven by development, environmental exposures, and lifestyle. These clocks further demonstrate that adult exposures can accelerate biological aging, linking lifestyle and environmental factors to age-related morbidity [94,95,96]. Dietary patterns, such as adherence to the Mediterranean diet, have also been associated with favorable methylation profiles and improved health outcomes [97].

It is important to note that phenotypic plasticity is itself an evolved trait shaped and constrained by genetic architecture. Each trait responds to environmental variation within a genetically encoded reaction norm. When environmental inputs fall outside this evolved reaction norm, because change is too rapid or too novel, plastic responses become limited or even maladaptive, contributing to developmental or adult mismatch. Recognizing these genetic constraints is essential for understanding the boundaries of plastic adaptation and its role in shaping vulnerability to modern NCDs.

### 4.2. Transgenerational Epigenetic Inheritance: Promises and Controversies

A critical question is whether environmentally induced epigenetic modifications are confined to individual lifetimes or whether they can persist across multiple generations. In mammals, epigenetic reprogramming during gametogenesis and early embryogenesis should erase most acquired marks [98]. Nevertheless, certain loci, including imprinted genes and transposable elements, may evade reprogramming, preserving alterations induced by diet, stress, or toxins [99,100,101].

The most robust demonstrations of TEI come from non-mammalian systems [102,103,104], whereas evidence in mammals, and especially in humans, is less consistent and often resolves into intergenerational associations rather than strict TEI beyond F2 [100,105,106]. Examples include the Dutch Hunger Winter [79], Holocaust survivor offspring [107], and recent work on Syrian refugees [108]. Despite these findings, the concept of true TEI in mammals remains controversial [100,106].

Nevertheless, rodent studies demonstrate that endocrine disruptors [109], paternal dietary exposures [110,111,112], paternal stress [113], and plastics [114] can induce epigenetic changes transmitted across generations. These findings suggest that while rare and mechanistically constrained, TEI is both biologically plausible and ecologically significant.

Emerging observations raise the intriguing possibility that TEI itself may contribute to maladaptation: a “transgenerational plasticity mismatch”, where inherited epigenetic states calibrated to past environments become liabilities under novel conditions. This issue intersects with the DOHaD framework and remains a key research frontier (Figure 1) [100,115].

It is essential to note that robust evidence for true transgenerational epigenetic inheritance (TEI) in humans remains scarce and highly debated. In mammals, extensive epigenetic reprogramming during gametogenesis and early embryogenesis poses a major mechanistic barrier to the persistence of acquired marks beyond F1. Moreover, in human populations, apparent multigenerational patterns are difficult to disentangle from powerful confounding mechanisms such as shared environments, socioeconomic transmission, cultural inheritance, and assortative mating [116,117,118,119,120,121,122,123,124]. These considerations imply that TEI in humans should be regarded as a plausible, but still unproven, contributor to disease risk rather than an established mechanism.

### 4.3. Epigenetics and the Evolution of the Genome

A growing body of evidence reveals that epigenetic mechanisms are not merely transient regulators of gene expression but also active drivers in genome evolution. DNA methylation, histone modifications, and chromatin architecture collectively influence mutation rates, recombination patterns, and the long-term stability of genetic information.

DNA methylation exerts a dual evolutionary role: it safeguards genome integrity by silencing transposable elements and repetitive sequences, yet intermittent loss of methylation permits their mobilization, providing bursts of regulatory and structural innovation that can be co-opted by natural selection [125,126,127]. This dynamic equilibrium between repression and release has profoundly shaped vertebrate genome architecture. Furthermore, methylated cytosines are inherently unstable, undergoing spontaneous deamination to thymine, thereby creating mutational hotspots that accelerate CpG→TpG transitions and contribute disproportionately to coding and regulatory sequence evolution [128,129].

Chromatin organization also constrains and channels recombination landscapes. Open, transcriptionally active domains often exhibit higher recombination rates, while heterochromatic regions remain recombinationally inert [130]. Such biases can influence linkage disequilibrium, promote adaptive haplotype blocks, and, over macroevolutionary timescales, affect karyotype evolution and speciation [131]. The structural plasticity of chromatin thus acts as an interface between epigenetic state and genome architecture.

Importantly, epigenetic variation can precede and bias genetic change, a process often termed “epigenetic priming.” Transiently induced epialleles—methylation-dependent regulatory states that arise in response to environmental stressors—can alter gene expression in ways that expose new phenotypic variation to selection. Over evolutionary time, such epigenetically mediated traits may undergo genetic assimilation, whereby initially reversible regulatory changes become fixed through sequence mutations [132,133]. This mechanism, first postulated by Waddington, represents a bridge between plasticity and evolution: short-term adaptive responses can sculpt long-term genomic landscapes.

Recent comparative genomics and experimental evolution studies further indicate that epigenetic responsiveness itself is an evolvable trait. Species occupying variable environments tend to retain higher epigenetic plasticity, suggesting a selective balance between stability and flexibility in epigenomic regulation. In this view, the epigenome functions as both a sensor and a memory of ecological change—transducing environmental inputs into molecular variation that can eventually be “hardwired” into the genome.

Together, these findings redefine the relationship between epigenetics, mutation, and selection, positioning epigenetic regulation not only as a mediator of phenotypic plasticity but also as a source of evolutionary innovation. The interplay between methylation dynamics, chromatin state, and genome architecture exemplifies how molecular mechanisms originally evolved for homeostatic regulation have become integral to the evolutionary process itself.

## 5. Mitigation Strategies: From Individual Behavior to Urban Health Policies

### 5.1. The Sustainability Challenge: Why Prevention Matters

The rapid increase in life expectancy during the twentieth century, while one of humanity’s greatest achievements, has created a paradox for health systems worldwide. As more people survive into advanced age, the prevalence of NCDs such as cardiovascular disease, diabetes, cancers, and neurodegenerative disorders has risen sharply, exerting unprecedented pressure on national healthcare systems. From an evolutionary perspective, this surge reflects a profound mismatch: physiological systems shaped under conditions of shorter lifespans, lower caloric intake, and higher pathogen exposure are now confronted with environments of longevity, energy abundance, and reduced infection burden, amplifying vulnerability to chronic disease [76].

This challenge is compounded by the demographic realities of many high-income countries, where population aging coincides with declining fertility rates. In such contexts, fewer working-age individuals support larger elderly populations, intensifying the financial and organizational burden on both public and private health systems [134].

Traditional models of care, centered on acute treatment, are ill-suited to chronic, lifestyle-related conditions that accumulate over decades. For this reason, primary prevention emerges as a critical strategy: rather than relying exclusively on increasingly expensive treatments in aging populations, societies must invest in reshaping environments to reduce risk at the source. Without such a shift, the sustainability of healthcare provision will be jeopardized, regardless of whether the system is funded publicly or privately.

### 5.2. Individual-Level Strategies: Aligning Daily Life with Human Biology

**Figure 3:**
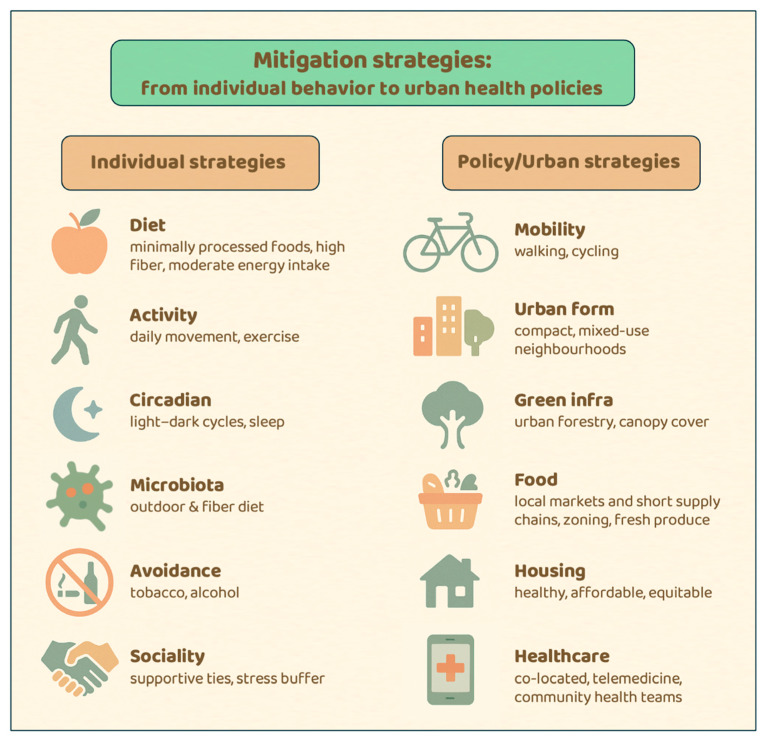
Mitigation strategies linking individual behaviors and urban/policy-level interventions. The figure illustrates how personal lifestyle choices and structural determinants interact across scales to reduce mismatch-related risks and improve population health.

At the level of individual behavior, prevention means restoring key elements of the ecological niches for which humans are biologically tuned. Evidence from evolutionary medicine and mismatch theory highlights several domains where realignment can mitigate NCD risk (Figure 3) [121].

Dietary patterns emphasizing minimally processed, nutrient-dense foods, high fiber, and moderate energy intake reduce the chronic anabolic signaling and glycemic volatility typical of ultra-processed diets; from the mismatch perspective, they recreate conditions of moderate abundance rather than continual excess [22,84,135,136,137].

Integrating frequent low- to moderate-intensity movement throughout the day, supplemented by occasional vigorous activity, reflects ancestral activity patterns and counters sedentary lifestyles [138,139,140]. Daily exposure to bright light in the morning and darkness at night stabilizes circadian clocks that regulate metabolism, immune tone, and sleep architecture, mitigating the consequences of artificial light at night and social jet lag [23,24]. Regular contact with biodiverse outdoor environments and diverse, fiber-rich diets supports immune education via the microbiota, buffering against allergic and inflammatory conditions [26,122]. Minimizing exposure to tobacco smoke and alcoholic beverages is essential. These exposures are evolutionarily novel, deliver toxins at doses and routes for which our lineage has had little time to evolve protective adaptations, and thus display steep dose–response gradients [3,141,142,143]. Finally, adequate, regular sleep and strong, supportive social ties dampen HPA-axis activation and other stress responses calibrated for small, cooperative groups, thereby reducing the chronic allostatic load characteristic of modern settings [144,145,146,147].

Notably, these practices are most effective when pursued together, because diet, activity, circadian timing, microbial exposures, sleep, and sociality are biologically intertwined. Yet framing them solely as matters of personal responsibility obscures the structural determinants of choice. The feasibility of realignment is shaped by factors such as the built environment, transport systems, food retail ecology, work schedules, housing quality, and access to green space and preventive care [121]. Accordingly, individual prevention must be coupled to population-level interventions and urban policies.

### 5.3. Policy-Level Strategies: Urban Health as Evolutionary Realignment

Cities, where the majority of the global population now resides, concentrate obesogenic food environments, physical inactivity, circadian disruption, pollution, and social isolation [148]. Yet they are also powerful levers for prevention: by redesigning urban environments, governments can make health-compatible behaviors the path of least resistance (Figure 3).

Mobility is a first lever: dense, connected walking and cycling networks, seamless public transport, and street designs that de-prioritize private cars increase daily physical activity while cutting air pollution and greenhouse-gas emissions [149].

Compact, mixed-use neighborhoods characterized by higher residential density, land-use mix, and proximate destinations increase walking and cycling, reduce car dependence, and lower cardiometabolic risk [149,150]. Combining compactness, connectivity, and active transport operationalizes “walkability” at scale [151,152,153].

Urban forestry functions as health-and-climate infrastructure: by shading sidewalks, lowering radiant temperature, and improving air quality, trees increase walkability, support well-being, and mitigate heat-island effects [154,155,156,157]. Ensuring equitable canopy distribution amplifies benefits in disadvantaged areas [158,159,160]. The food environment can be shifted by supporting peri-urban agriculture, local markets, and regulating fast-food clustering, nudging diets away from ultra-processed products [161,162,163]. On the housing and equity front, smoke-free, ventilated, thermally adequate dwellings, coupled with anti-segregation and affordability policies, reduce exposure to pollutants and crowding while protecting vulnerable groups [164].

Finally, integrated healthcare access—through co-located primary care, telemedicine, and proactive community health teams—improves continuity of care and preventive service delivery [164].

### 5.4. Governing Complexity: Towards a New Urban Health Science

Urban health cannot be reduced to isolated interventions. Cities are socio-ecological–technological systems (SETS) where transport, housing, economy, and culture interact in non-linear ways. Governance models that are polycentric, adaptive, and participatory can better address this complexity [165,166].

The goal is not to recreate a Paleolithic utopia, but to design forward-looking environments that respect human biology. By embedding movement, rhythms, diverse diets, and social connectedness into urban form, we can mitigate mismatches, reduce NCD burdens, and ensure sustainable healthcare.

## 6. Conclusions and Future Perspectives

The contemporary health landscape is shaped by a paradox. On the one hand, modern societies have extended human life expectancy to unprecedented levels; on the other, this success has exposed our species to a heavy burden of non-communicable diseases. From an evolutionary perspective, these conditions are not anomalies but predictable outcomes of trade-offs, constraints, and mismatches between our evolved biology and our rapidly changing environments.

The theory of evolutionary mismatch provides a unifying framework to explain why Homo sapiens has become an “evolutionary misfit.” Our genomes were sculpted in ecological niches of scarcity, microbial diversity, and natural rhythms, yet we now inhabit urbanized, sanitized, sedentary, and resource-abundant settings. Developmental perspectives add further nuance: early-life plasticity offers predictive adjustments, but when adult conditions diverge, developmental mismatches ensue. Physiological systems that evolved for acute, fluctuating challenges, like stress responses, circadian clocks and inflammatory pathways, are today chronically activated, amplifying vulnerability.

The rise of non-communicable diseases must also be understood through the lens of aging. Mutation accumulation, antagonistic pleiotropy, and the disposable soma theory explain why selection allows late-life decline. The demographic transition to modernity has not created aging but has made its consequences universal, turning chronic disease into the dominant public health challenge.

Confronted with these realities, the sustainability of healthcare systems, whether publicly or privately funded, depends increasingly on prevention. Populations are aging, fertility rates are declining, and the costs of treating chronic diseases are rising unsustainably. Primary prevention, informed by evolutionary and developmental insights, is no longer optional but essential. Several lines of evidence indicate that, at the individual level, realigning daily life with our biology through diet, movement, circadian hygiene, microbial exposures, and social connectedness can buffer these mismatches. Yet the greatest leverage lies in systemic change. Urban health policies, grounded in a new science of complex socio-ecological–technological systems, offer pathways to recreate environments that support, rather than undermine, human biology.

Despite substantial advances, several major questions remain open. First, the limits and evolutionary constraints of human plasticity, including the shape and evolvability of reaction norms, require mechanistic clarification. Second, distinguishing genuine TEI from cultural or environmental transmission demands longitudinal, multi-generational designs combined with molecular evidence. Third, more integrative frameworks are needed to connect short-term epigenetic responsiveness with long-term evolutionary processes. Finally, urban health interventions inspired by evolutionary principles require prospective experimental evaluation to refine and optimize their implementation in real-world contexts. Addressing these questions will deepen the theoretical foundations of evolutionary medicine and inform more effective prevention strategies.

Although many aspects remain to be clarified, it should be considered that this narrative, grounded in robust scientific evidence, may enhance the uptake of health-promoting behaviors by individuals and the adoption of supportive governance choices, leveraging the well-documented appeal that ‘ancestral’ lifestyles exert on a sizeable segment of the population. However, the challenge is not to return nostalgically to a mythical past, but to design forward-looking societies that respect our evolutionary heritage while embracing modern innovation. If we succeed, we may turn the label of the “evolutionary misfit” from a diagnosis of vulnerability into a roadmap for resilience.

## Data Availability

No new data were created or analyzed in this study. Data sharing is not applicable to this article.

## Abbreviations

The following abbreviations are used in this manuscript:

DOHaD	Developmental origins of health and disease
DTA	Developmental theory of aging journals
HPA	Hypothalamic–pituitary–adrenal
NCDs	Non-communicable diseases
SETS	Socio-ecological–technological systems
TEI	Transgenerational epigenetic inheritance
